# Real‐World Data on the p.Gly17Val RHOA Mutation in Diagnosing T Follicular Helper Cell Lymphomas

**DOI:** 10.1002/cam4.70955

**Published:** 2025-05-10

**Authors:** Yuri Tsuboi, Keiichiro Hattori, Naoki Kurita, Yasuhito Suehara, Toru Nanmoku, Ryota Matsuoka, Ryota Ishii, Sakurako Suma, Kenichi Makishima, Yumiko Maruyama, Tatsuhiro Sakamoto, Takayasu Kato, Hidekazu Nishikii, Naoshi Obara, Daisuke Matsubara, Mamiko Sakata‐Yanagimoto

**Affiliations:** ^1^ Department of Hematology University of Tsukuba Hospital Tsukuba Ibaraki Japan; ^2^ Department of Hematology Graduate School of Comprehensive Human Sciences, University of Tsukuba Tsukuba Ibaraki Japan; ^3^ Department of Hematology Institute of Medicine, University of Tsukuba Tsukuba Ibaraki Japan; ^4^ Department of Clinical Laboratory University of Tsukuba Hospital Tsukuba Ibaraki Japan; ^5^ Department of Pathology Institute of Medicine, University of Tsukuba Tsukuba Ibaraki Japan; ^6^ Department of Biostatistics Institute of Medicine, University of Tsukuba Tsukuba Ibaraki Japan; ^7^ Division of Advanced Hemato‐Oncology Transborder Medical Research Center, University of Tsukuba Tsukuba Ibaraki Japan

**Keywords:** angioimmunoblastic T‐cell lymphoma, G17V *RHOA* mutation, T follicular helper cell lymphoma

## Abstract

**Background:**

T follicular helper (Tfh) cell lymphomas, including their most prevalent form, angioimmunoblastic T‐cell lymphoma, frequently present with clinical symptoms, such as fever and rash, accompanied by substantial immune cell infiltration within the tumor microenvironment. These features often obscure the distinction between Tfh lymphoma and other autoimmune or inflammatory conditions. Notably, the p.Gly17Val *RHOA* (G17V) mutation is commonly associated with Tfh lymphomas, including angioimmunoblastic T‐cell lymphoma, suggesting that testing for the G17V mutation may serve as a valuable diagnostic tool. However, it remains unclear which patients would benefit the most from G17V mutation testing.

**Methods:**

In this study, we retrospectively reviewed the medical records of 224 patients tested for the G17V mutation as part of routine clinical practice.

**Results:**

We detected G17V in 17 patients. Among Tfh lymphoma cases, the sensitivity and specificity of the G17V test were 0.533 and 0.955, respectively. We further explored the association between G17V positivity and the clinical features of patients undergoing testing for the first time (*n* = 186). The G17V mutation was more frequent in patients presenting with lymphadenopathy in combination with fever or skin rash and elevated soluble interleukin‐2 receptor levels (*p* = 0.002). The median time from G17V test submission to results was 11.6 d shorter than that for pathological diagnosis (*p* = 0.0009).

**Conclusions:**

Given the noninvasive nature of the G17V test, its rapid administration to appropriate patients is expected to enable faster and more efficient diagnosis of Tfh lymphomas compared with conventional methods.

## Graphical abstract

Tfh lymphomas, including angioimmunoblastic T‐cell lymphoma, often present with symptoms resembling autoimmune conditions. The p.Gly17Val RHOA (G17V) mutation is commonly found in these lymphomas and can aid diagnosis, offering faster results, especially in patients with specific clinical features like lymphadenopathy, fever, and skin rash.

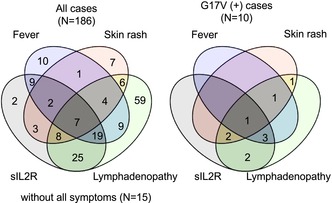

**Abbreviations**
AITL: Angioimmunoblastic T‐cell lymphoma
ddPCR: Droplet digital polymerase chain reaction
FFPE: formalin‐fixed paraffin‐embedded
G17V: p.Gly17Val *RHOA* mutation
LDH: lactate dehydrogenase
PCR: polymerase chain reaction
PTCL‐NOS: nodal peripheral T‐cell lymphoma not otherwise specified
PTCL‐Tfh: nodal peripheral T‐cell lymphoma with a T follicular helper phenotype
sIL2R: soluble interleukin‐2 receptor
Tfh: T follicular helper
VAF: variant allele frequency

## 1 Introduction

Angioimmunoblastic T‐cell lymphoma (AITL), nodal peripheral T‐cell lymphoma with a T follicular helper (Tfh) phenotype (PTCL‐Tfh), and follicular T‐cell lymphoma, which are classified as Tfh cell lymphomas, are characterized by the expression of marker proteins similar to those found in Tfh cells [1]. AITL occurs mainly in older patients and has a poor prognosis, with a 5‐year survival rate of 30% to 40% [1]. The clinical features of AITL include lymphadenopathy, high fever, skin rash, fluid retention, and autoimmune‐like manifestations, such as arthritis [2, 3, 4]. These symptoms make distinguishing AITL from other autoimmune disorders or inflammatory conditions difficult. Laboratory findings, such as high levels of lactate dehydrogenase (LDH) and soluble interleukin‐2 receptor (sIL2R), hypergammaglobulinemia, and a positive Coombs' test in the serum, are commonly observed, further complicating the diagnosis [5]. AITL is pathologically characterized by the disruption of follicular structure and diverse cellular infiltrates, including lymphoma cells with clear cytoplasm, immunoblasts, plasma cells, and follicular dendritic cells [6, 7]. However, in its early stages, the disease is characterized by preserved lymph node architecture with abundant hyperplastic lymphoid follicles and difficulty in identifying malignant lymphoma cells [8], making the diagnosis of AITL particularly challenging.

It is noteworthy that the G17V *RHOA* mutation (G17V) is specifically identified in 53%–68% of cases of AITL and other Tfh lymphomas, such as PTCL‐Tfh and follicular T‐cell lymphoma [9, 10, 11, 12], while its occurrence is exceedingly rare in other hematologic malignancies [9]. Since AITL is often difficult to diagnose based only on clinical manifestations and pathological findings, G17V testing is expected to provide valuable support for diagnosis. We developed a detection system for G17V by optimizing the allele‐specific polymerase chain reaction (PCR) method, which was previously reported in 2014 [13], and adapted it to the WAVE system [14]. This system has been used as an in‐house diagnostic assay for G17V mutation since 2016. We conducted tests on cases requiring differential diagnosis of fever of unknown origin and other conditions, ultimately performing examinations on cases involving a variety of diseases for the final diagnosis. The mechanism of the WAVE system is based on the denaturing high‐performance liquid chromatography method, in which both wild‐type and mutant samples are amplified by allele‐specific PCR, and the mutation ratio is detected by comparing the heights of the waveforms of each sample using denaturing high‐performance liquid chromatography.

Although G17V testing may be useful in addressing the diagnostic challenges of AITL, it is not clear which patients should undergo G17V testing. Therefore, this study was conducted to identify the clinical characteristics of patients who underwent G17V testing, as well as those with confirmed positive or negative G17V results, and to determine which patient profiles would benefit the most from proactive G17V mutation testing.

## 2 Methods

### 2.1 WAVE System

Genomic DNA (50 ng) was subjected to PCR using EX‐Taq HS (TaKaRa Bio). The following primers were used: *RHOA*‐Wildtype‐Forward 5′‐ATTGTTGGTGATGGAGCCTGAGG‐3′, *RHOA*‐Mutant‐Forward 5′‐ATTGTTGGTGATGGAGCCTGAGT‐3′, and *RHOA*‐Reverse (RHOA‐R) 5′‐ACACCTCTGGGAACTGGTCCT‐3′. The primer pairs *RHOA*‐Wildtype‐Forward and RHOA‐R were used to detect the wild‐type allele, whereas *RHOA*‐Mutant‐Forward and RHOA‐R were used to detect the mutant allele. The amplification protocol consisted of 35 cycles of 94°C for 30 s, 66°C for 30 s, and 72°C for 1 min. The PCR products (6.5 μL/sample) were visualized on a 2.5% agarose gel stained with ethidium bromide. In samples where a band corresponding to the mutant allele was observed, the PCR product (10 μL/sample) for both wild‐type and mutant alleles was analyzed by the WAVE system to calculate the percentage of the mutant allele based on the height of the waveform.

### 2.2 Droplet Digital PCR (ddPCR) Assays

We performed ddPCR using the QX200 Droplet Digital PCR system (Bio‐Rad), following the protocol previously described by Nuhat et al. [15].

### 2.3 Clinical Information

The study was approved by the Ethics Committee of the University of Tsukuba Hospital (approval numbers: H24‐74 and R06‐28). We enrolled all patients who underwent G17V mutation analysis using the WAVE system at the University of Tsukuba Hospital between June 2016 and November 2023. In total, 233 patients were included in the study. Nine patients were excluded due to insufficient clinical information. The clinical and laboratory characteristics of the remaining 224 patients were retrospectively analyzed (Table S1). Patients were diagnosed according to the 2016 World Health Organization criteria. In this cohort, 186 patients underwent G17V mutation analysis for the first time and had sufficient clinical data available for analysis (Table S2). Clinical parameters were extracted from the medical records, including three clinical manifestations (fever: body temperature ≥ 37.5°C, skin rash, and lymphadenopathy) and two laboratory data points (LDH and sIL2R levels).

### 2.4 Samples

The sample types of the 224 patients were as follows: lymph node (*n* = 105), bone marrow (*n* = 63), liquid (*n* = 29, 6 of there were also analyzed using lymph node or bone marrow samples), skin (*n* = 10), FFPE (*n* = 4), and others (*n* = 19) (Table S1).

### 2.5 Statistical Analysis

Receiver operating characteristic curve analysis was used to determine the cut‐off values for LDH and sIL2R, which are both predicted to have a relation with G17V positivity. The relation between clinical characteristics and G17V positivity was evaluated by logistic regression. Among clinical characteristics, only lymphadenopathy was analyzed using Firth's penalized logistic regression due to complete separation. Clinical characteristics were compared using logistic regression analysis. The t‐test was used to compare the median duration from the initial consultation to the receipt of the G17V test results and pathological findings. All analyses were performed using R software [16]. A *p*‐value < 0.05 was considered statistically significant.

## 3 Results

### 3.1 G17V Detection Sensitivity Using the WAVE System

**FIGURE 1:**
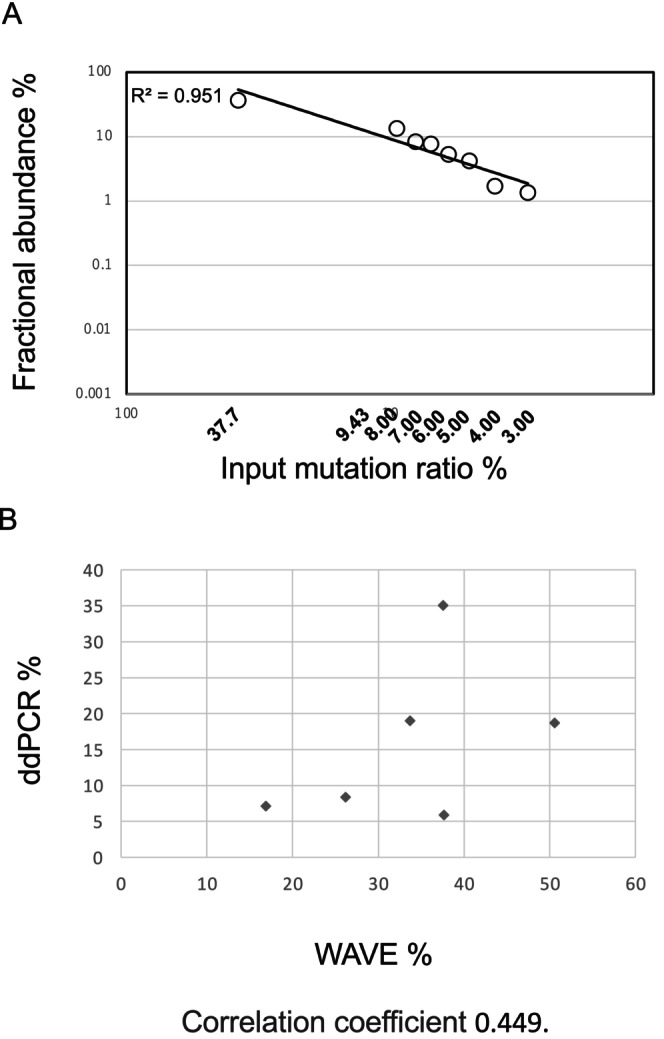
Detection of the p.Gly17Val *RHOA* mutation (G17V) using the WAVE system. (A) Sensitivity of G17V detection using the WAVE system. (B) Comparison of the p.Gly17Val *RHOA* mutation ratio detected by the WAVE system and droplet digital polymerase chain reaction (ddPCR). The *X*‐axis indicates the mutation ratio detected by the WAVE system, whereas the *Y*‐axis indicates the mutation ratio detected by ddPCR.

First, we investigated the lower limits of detection of the WAVE system. We prepared serial dilutions of G17V‐mutated genomic DNA in wild‐type DNA at variant allele frequency (VAF) levels of 37.7%, 9.43%, 8.00%, 7.00%, 6.00%, 5.00%, 4.00%, 3.00%, 2.36%, 0.59%, 0.15%, 0.04%, and 0.00%. G17V‐mutated genomic DNA was extracted from patient 205 and wild‐type DNA from patient 12 (Table S1). The mutation ratios detected using the WAVE system were 36.7%, 13.4%, 8.35%, 7.70%, 5.30%, 4.20%, 1.70%, and 1.35%, respectively. Samples diluted below 3.00% were undetectable, yielding a result of 0.00% (Figure 1A). In summary, the WAVE system detected G17V at dilutions as low as 3.00%, with a corresponding mutation ratio of 1.35%.

As previously reported, ddPCR is highly valuable for detecting G17V for research purposes [15]. To demonstrate the effectiveness of the WAVE system in detecting G17V across various samples, we measured G17V in six samples (two lymph node samples [L1 (patient 159) and L2 (patient 205)], two plasma samples [P1 (patient 12) and P2 (patient 159)], and two formalin‐fixed paraffin‐embedded (FFPE) samples [F1 (not included in this study cohort) and F2 (not included in this study cohort)]) using the WAVE system and ddPCR assay as controls. The mutation ratios detected by the WAVE system and ddPCR were as follows: L1, 50.6%, 23.10%; L2, 37.70%, 5.94%; P1, 33.7%, 19.0%; P2, 37.6%, 35.07%; F1, 16.9%, 7.18%; F2, 26.2%, 8.38% (Figure 1B). The correlation coefficient between the mutation ratios detected using ddPCR and WAVE was 0.449 (Figure 1B). The WAVE system was able to detect G17V in different types of samples.

### 3.2 Relation Between G17V Positivity and Clinical Characteristics

**FIGURE 2:**
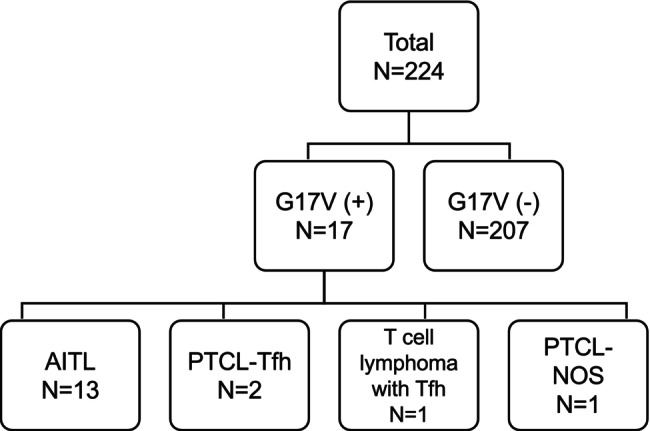
Summary of 224 cases enrolled in this study. AITL, angioimmunoblastic T‐cell lymphoma; PTCL‐Tfh, nodal peripheral T‐cell lymphoma with a T follicular helper phenotype; PTCL‐NOS, nodal peripheral T‐cell lymphoma, not otherwise specified; G17V, p.Gly17Val *RHOA* mutation.

We enrolled 224 patients with available nodal or extranodal samples and clinical records. In most patients, the sample used for G17V mutation analysis was fresh tissue, but four patients (patients 99, 119, 153, and 214) used FFPE samples. Patient information, including the final diagnoses, is presented in Table S1. Based on the final diagnosis, 30 patients had Tfh lymphoma, including AITL (*n* = 25), PTCL‐Tfh (*n* = 3), T‐cell lymphoma with Tfh phenotype (*n* = 1, only bone marrow specimen was available), and follicular T‐cell lymphoma (*n* = 1). Besides, 19 patients had PTCL not otherwise specified (PTCL‐NOS). Among them, G17V was detected in 17 patients: AITL (*n* = 13), PTCL‐Tfh (*n* = 2), T‐cell lymphoma with Tfh phenotype (*n* = 1), and PTCL‐NOS (*n* = 1) (Figure 2). The sensitivity and specificity of the G17V test using the WAVE system for patients with Tfh lymphoma were 0.533 and 0.995, respectively.

Regarding the G17V‐negative patients, the breakdown of final diagnoses was as follows: non‐Tfh T‐cell lymphoma (*n* = 36), fever of unknown origin or reactive/atypical lymphoid hyperplasia or inflammation (*n* = 26), diffuse large B‐cell lymphoma (*n* = 22), collagen‐ or immune‐related diseases (*n* = 22), AITL (*n* = 12), carcinoma (*n* = 11), PTCL‐Tfh (*n* = 1), follicular T‐cell lymphoma (*n* = 1) and others (*n* = 76).

**FIGURE 3:**
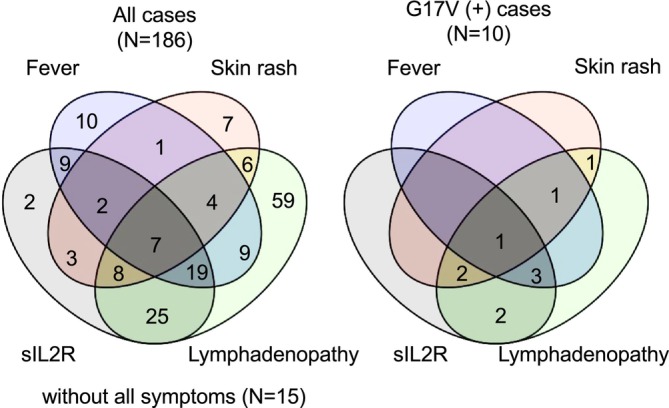
Relation between the p.Gly17Val *RHOA* mutation and clinical manifestations. sIL2R, soluble interleukin‐2 receptor; G17V, p.Gly17Val *RHOA* mutation.

Based on the receiver operating characteristic curve analysis, the cut‐off values were 289.5 U/L and 2295 U/mL for LDH and sIL2R, respectively. Of these, G17V positivity was more strongly associated with sIL2R (*p* = 0.02) than with LDH (*p* = 0.1). Therefore, we focused on the relation between G17V positivity, three clinical symptoms (fever, skin rash, and lymphadenopathy), and one laboratory data point (sIL2R) in patients who underwent G17V testing for the first time (*n* = 186) (Table 1). The Venn diagram illustrates the association between G17V positivity and these clinical parameters (Figure 3). All 10 G17V‐positive patients presenting with lymphadenopathy were accompanied by additional characteristic symptoms or laboratory findings. Further analysis revealed that among individual factors, skin rash was most strongly associated with G17V positivity (*p* = 0.027) (Table 1). The combination of lymphadenopathy and either or both fever and skin rash was significantly correlated with G17V positivity (*p* = 0.002) (Table 1). Furthermore, patients with multiple characteristic factors, such as lymphadenopathy and sIL2R, along with either or both fever and skin rash, exhibited higher positivity for G17V (*p* = 0.002) (Table 1).

**TABLE 1:** Relation between clinical data and p.Gly17Val *RHOA* mutation positivity.

Clinical symptoms				p.Gly17Val *RHOA* mutation		OR (95% CI)	*p*
Fever	Skin rash	LN	sIL2R	Positive, *N* (%)	Negative, *N* (%)		
				*N* = 10	*N* = 176		
●				5 (50)	56 (32)	2.14 (0.57–7.99)	0.24
	●			5 (50)	33 (19)	4.33 (1.15–16.4)	0.027
		●		10 (100)	127 (72)	8.15 (1.02–1056)	0.048
			●	8 (80)	67 (38)	6.51 (1.57–44.0)	0.02
●	●			2 (20)	12 (6.8)	3.42 (0.48–15.6)	0.15
●			●	4 (40)	33 (19)	2.89 (0.71–10.7)	0.12
	●		●	3 (30)	17 (9.7)	4.01 (0.81–16.0)	0.059
		●	●	8 (80)	51 (29)	9.8 (2.36–66.5)	0.005
●	●		●	1 (10)	8 (4.5)	2.33 (0.12–14.9)	0.45
Either or both		●		8 (80)	45 (26)	11.6 (2.79–79.1)	0.002
Either or both		●	●	6 (60)	28 (16)	7.93 (2.13–32.8)	0.002

Abbreviations: 95% CI, 95% confidence interval; NA, not available.

Among the 12 cases initially identified as G17V‐negative AITL, we performed ddPCR analysis on 10 cases for which additional samples were available (Table S3). G17V mutations were detected in patient 53 and patient 125 at variant allele frequencies of 0.05% and 0.72%, respectively. As expected, in cases with a low mutation burden, the WAVE method may yield false‐negative results.

Moreover, as patient 125 was a newly diagnosed case, we reanalyzed the association between clinical features and the presence of the G17V mutation (Table S4). The consistent result was obtained as in the analysis based on the WAVE system. Skin rash was strongly associated with G17V positivity (*p* = 0.008). The combination of lymphadenopathy with either or both fever and skin rash was also significantly linked (*p* = 0.001), and multiple factors, such as lymphadenopathy and sIL2R, along with either or both fever and skin rash, further increased G17Vpositivity (*p <* 0.001) (Table S4).

### 3.3 G17V Detection by Liquid Biopsy

A total of 29 patients underwent liquid biopsy, demonstrating a sensitivity of 0.75 and a specificity of 0.96. Among the G17V‐positive cases identified by tissue specimens (*n* = 17), liquid biopsy was performed in six cases, including plasma (*n* = 4), pericardial effusion (*n* = 1) (previously reported Ref. [17]), and ascites (*n* = 1). For the four patients who underwent G17V testing for both plasma and lymph node, G17V was detected in all samples (mutant ratio in plasma, range 5.1%–37.6%; lymph node, range 35.4%–50.6%). Patients whose pericardial or ascitic fluid was positive for G17V did not have lymph nodes available for G17V testing because of the absence of lymphadenopathy, although their blood and bone marrow were positive for G17V. These findings suggest that the sensitivity of G17V detection by liquid biopsy using the WAVE system is highly feasible.

### 3.4 Time Required For the G17V Test or Pathological Diagnosis

**FIGURE 4:**
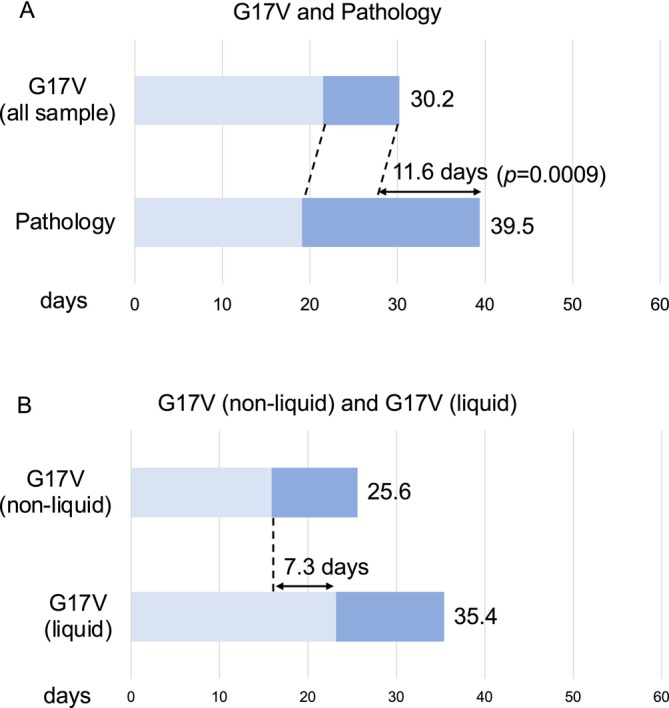
Duration from consultation or test submission to receiving the results of the p.Gly17Val *RHOA* mutation test or pathology. (A) Time course difference between G17V testing and pathology in all samples. The upper bar shows G17V testing, and the lower bar shows pathology. Light blue bars indicate the days from consultation to G17V or pathology submission, and dark blue bars indicate the days from G17V or pathology submission to results identification. (B) Time course differences in G17V testing between non‐liquid (lymph node, skin, bone marrow, and blood) samples and liquid (plasma, pericardial effusion, and ascites) samples. The upper bar shows non‐liquid samples, and the lower bar shows liquid samples. Light blue bars indicate the days from consultation to G17V submission, and dark blue bars indicate the days from G17V submission to results identification. G17V, p.Gly17Val *RHOA* mutations.

For G17V‐positive cases (*n* = 17), we examined the time required from either the first consultation: the date of the initial visit to the hematology outpatient clinic or submission of the test: the first date on which the G17V test was submitted at our hospital to the receipt of results for both the G17V test and pathological diagnosis (Figure 4A). The duration from the first consultation to receipt of the results of the G17V test (median 30.2 d, range 9–77) was shorter than that of the pathological diagnosis (median 39.5 d, range 19–61, *p* = 0.185) (Figure 4A). Notably, the duration from test submission to receipt of the results of the G17V test (median 8.7 d, range 1–21) was 11.6 d shorter than that of the pathological diagnosis (median 20.3 d, range 6–49, *p* = 0.0009), whereas the duration from consultation to test submission was not different between the G17V test and pathological diagnosis (Figure 4A).

Subsequently, we compared the time required for the return of results for various samples subjected to the G17V test (Figure 4B). The samples were categorized into two groups: non‐liquid (lymph nodes, skin, bone marrow, and blood) and liquid (plasma, pericardial effusion, and ascites). Although there was no significant difference in the days from consultation to receipt of results between the non‐liquid group (median 25.6 d, range 9–77) and the liquid group (median 35.4 d, range 14–74, *p* = 0.269), the liquid group submitted the test 7.3 d later than the non‐liquid group (Figure 4B).

## 4 Discussion

In the present study, we clarified the characteristics of patients with positive G17V test results by analyzing real‐world clinical test data using the WAVE method. The positivity rate of G17V in patients with AITL and other Tfh lymphomas using the WAVE system was consistent with previous reports [9, 10, 11]. In clinical practice, it is preferable to test populations with high pre‐test probabilities. Real‐world data from our hospital indicated that, as an individual factor, the presence of skin rash was most strongly associated with G17V positivity. Furthermore, there is a strong association between G17V positivity and a combination of clinical symptoms, such as fever, skin rash, lymphadenopathy, and sIL2R. Therefore, it is important to consider the multiple AITL‐related clinical and laboratory factors that determine the likelihood of mutation positivity.

Various methods have been used to detect the G17V mutation (Table 2). Although the WAVE system has lower detection sensitivity than ddPCR (detection limit: WAVE 3.00% vs. ddPCR 0.04%) and other approaches, including next‐generation sequencing [13, 15], it offers advantages in terms of simplicity, versatility, and cost performance. These features facilitate its application in in‐hospital screening. Notably, AITL often exhibits a low tumor cell content; thus, the VAFs of G17V in AITL samples have been reported to range from 0.40% to 45.3% [9, 12, 18, 19, 20, 21, 22, 23]. Among patients with G17V‐positive AITL, those with a higher VAF, which reflects a high tumor burden, tend to have a worse prognosis [24]. Based on our findings, the WAVE system can reliably identify cases with relatively high VAF values, which are associated with a poor prognosis. Especially when the biopsy specimen is small and limited, confirming the G17V mutation can be useful for diagnosis. For cases with a low tumor ratio, more sensitive assays may be necessary for an accurate diagnosis.

**TABLE 2:** Summary of detection methods for p.Gly17Val *RHOA* mutation.

Author	Detecting methods	Sample types	Median VAF% (range)
Hsu [18]	Deep sequencing	FFPE	NA
Miyoshi [12]	Deep sequencing Sanger sequencing	FFPE	14.9 (4.0–25.9)
Lee [19]	Cast‐PCR	FFPE, FNAC	11.8 (2.0–30.6)
Stainhilber [20]	NGS	FFPE	8.0 (1.0–31.0)
Nuhat [15]	ddPCR NGS PNA‐LNA clamp method	Frozen specimens	NA
Sakata‐Yanagimoto Nakamoto‐Matsubara [21]	Targeted sequencing	Serum Frozen specimens FFPE	9.8 (2.5–17.3)
Ondrejika [17]	Targeted exome sequencing Sanger sequencing	FFPE	14.0 (0.4–50)
Nagao [16]	Deep sequencing SNP genotyping Sanger sequencing	FFPE	8.0 (4.0–13.6)
Nakamoto‐Matsubara [13]	AS‐PCR	FFPE PLP‐fixed specimens Frozen specimens	NA
Sakata‐Yanagimoto [9]	Targeted deep sequencing	Frozen specimens	10.4 (2.96–45.3)

Abbreviations: AS‐PCR, allele‐specific polymerase chain reaction; Cast‐PCR, competitive allele‐specific TaqMan polymerase chain reaction; ddPCR, droplet digital polymerase chain reaction; FCM, flow cytometry; FFPE, formalin‐fixed paraffin‐embedded; FNAC, fine needle aspiration cytology; NA, not available; NGS, next‐generation sequencing; PLP, periodate/lysine/paraformaldehyde; VAF, variant allele frequency.

In this study, 20 patients tested for G17V for the first time (*n* = 186) were finally diagnosed with collagen‐ or immune‐related diseases that were difficult to distinguish from AITL and other Tfh lymphomas based on clinical findings. In particular, five patients were diagnosed with adult‐onset Still's disease, one patient only had fever, and the other four patients had fever with a combination of lymphadenopathy, skin rash, or elevated sIL2R. G17V testing may aid in the diagnosis of these conditions. Conversely, of the 24 patients with fever of unknown origin or reactive/atypical lymphoid hyperplasia or inflammation (*n* = 24), 12 had only a single symptom, such as fever or lymphadenopathy, and four had none. Nine patients with a final diagnosis of carcinoma presented only with lymphadenopathy, indicating a low pre‐test probability of G17V. These findings provide useful guidelines for G17V testing.

The duration from consultation to receiving the test results was significantly shorter for the G17V test than for pathology. Specifically, the duration from test submission to diagnosis was 11.6 d shorter, facilitating earlier diagnosis. This is particularly advantageous in facilities where a hematopathologist is not available. Moreover, the results of the G17V testing contributed to the diagnostic process, particularly in evaluating the presence of a Tfh phenotype or in cases where determining tumor infiltration in extranodal lesions was challenging. In these real‐world data, the submission of liquid specimens was delayed by 7.3 d compared with other sample types. This delay is likely due to the diagnostic difficulty of AITL and the clinical practice of submitting G17V testing along with lymph node biopsies, with liquid specimens submitted later in cases of suspected T‐cell lymphoma. Nevertheless, liquid samples, particularly plasma, are easier to obtain than lymph node tissues. Therefore, it is recommended to prioritize earlier G17V testing of liquid specimens in cases where AITL is suspected based on multiple characteristic clinical and laboratory factors. It allows for early intervention in patients with lymphadenopathy and immune‐related reactions who are in urgent need of treatment. Moreover, the availability of liquid biopsy enables early treatment of patients without lymphadenopathy, as a positive G17V result indicates a high probability of Tfh lymphoma.

This study has several limitations. The prevalence of AITL is low due to the rarity of the disease, and the number of G17V‐positive cases is limited. Consequently, when analyzing the relation between G17V and clinical and laboratory findings, overly restrictive parameters reduced the number of positive cases, resulting in a failure to detect significant differences. In addition, the retrospective analysis may have introduced selection bias, resulting in an enrichment of cases with suspected Tfh lymphoma.

In conclusion, this study demonstrated that the G17V test using the WAVE system is a reliable and feasible method for detecting mutations in AITL and other Tfh lymphomas. Understanding the patient profiles that are more likely to test positive for the G17V test and considering the appropriate circumstances for performing it will enhance the utility of the G17V test as a valuable diagnostic tool.

## Author Contributions

**Yuri Tsuboi:** data curation (lead), formal analysis (lead), writing – original draft (lead). **Keiichiro Hattori:** methodology (equal), supervision (lead), writing – review and editing (equal). **Naoki Kurita:** methodology (equal), supervision (lead), writing – review and editing (equal). **Yasuhito Suehara:** resources (equal), writing – review and editing (equal). **Toru Nanmoku:** methodology (equal), resources (equal). **Ryota Matsuoka:** validation (equal), writing – review and editing (equal). **Ryota Ishii:** methodology (equal), validation (equal). **Sakurako Suma:** writing – review and editing (equal). **Kenichi Makishima:** writing – review and editing (equal). **Yumiko Maruyama:** writing – review and editing (equal). **Tatsuhiro Sakamoto:** writing – review and editing (equal). **Takayasu Kato:** writing – review and editing (equal). **Hidekazu Nishikii:** writing – review and editing (equal). **Naoshi Obara:** writing – review and editing (equal). **Daisuke Matsubara:** writing – review and editing (equal). **Mamiko Sakata‐Yanagimoto:** conceptualization (lead), funding acquisition (lead), project administration (lead), writing – review and editing (lead).

## Ethics Statement

The study was approved by the institutional review board at the University of Tsukuba Hospital.

‐Informed Consent: N/A.

This is a study that is conducted using only information such as medical information, without any invasion or intervention with the patient, and it falls under the public health and academic exceptions of the Personal Information Protection Law.

For the above reasons, we have decided to handle this study as an opt‐out study.

‐Registry and the Registration Numbers: H24‐74 and R06‐28.

‐Animal Studies: N/A.

## Conflicts of Interest

The authors declare no conflicts of interest.

## Supporting information


**Table S1.** Patients included in this study.
**Table S2.** Patients who examined G17V RHOA mutation for the first time.
**Table S3.** ddPCR for G17V RHOA mutation negative cases.
**Table S4.** Relation between clinical data and p.Gly17Val RHOA mutation positivity, including patient 125.

## Data Availability

The data and materials are available upon request.
